# Model-based process optimization of black soldier fly egg production

**DOI:** 10.3389/fbioe.2024.1404776

**Published:** 2024-05-22

**Authors:** Alexander Kobelski, Arne-Jens Hempel, Murali Padmanabha, Patrick Klüber, Luiz-Carlos Wille, Stefan Streif

**Affiliations:** ^1^ Automatic Control and System Dynamics Lab, Technische Universität Chemnitz, Chemnitz, Germany; ^2^ Lab for Digital Engineering, Staatliche Studienakademie Glauchau, Glauchau, Germany; ^3^ Fraunhofer Institute for Molecular Biology and Applied Ecology, Department of Bioresources, Gießen, Germany

**Keywords:** black soldier fly (BSF), insect rearing, reproduction, egg production, control, modeling, automation

## Abstract

Black soldier fly (BSF) larvae (*Hermetia illucens*) serve as a valuable protein source for animal feed. Limiting factors in the industrial rearing of BSF are the reproduction process and egg output. Studies indicate the potential to shorten preoviposition time and increase egg output through better utilization of environmental variables, such as temperature and light, in industrial settings. Excessive stimulation, however, can lead to stress, elevated production costs, and reduced egg numbers, emphasizing the need for a delicate balance. This study addresses these challenges by investigating controlled manipulation of environmental variables to stimulate mating and enhance egg production, thereby developing a comprehensive model encompassing the adult fly life cycle, mating, and egg production. Model parameters were fitted using literature data, and the model’s plausibility was tested through simulations. Using the model and optimal control methods, the calculated dynamic trajectories for environmental variables when compared to the standard approach in a constant environment demonstrated higher output and shorter production cycles at reasonable energy costs. Applications for this model-based optimization are demonstrated for various scenarios, highlighting the practical utility and versatility of the developed model. This study contributes valuable insights for improving rearing practices of BSF through environmental stimulation, offering potential advancements in egg production efficiency and overall sustainability.

## 1 Introduction

Mass production of insects is a relatively young but rapidly evolving industrial sector which shows great potential for both standalone and circular production of high value proteins (Francuski and Beukeboom, 2020; Chavez, 2021). Efforts to understand and enhance mass rearing typically focus on investigating the effects of fixing parameters such as feed or temperature at a constant level (Nayak et al., 2024). While some aspects of automation have been investigated (Kröncke et al., 2020), there is limited work on dynamic modeling of insect rearing and subsequent dynamic process optimization and control Padmanabha et al. (2023).

Insect proteins from the black soldier fly (BSF) *Hermetia illucens* are a promising alternative to conventional animal proteins. The BSF larvae can feed on a variety of substrates such as animal feed, algae, or waste (Liland et al., 2017), which makes their rearing less dependent on global trade fluctuations. Surendra et al. (2020) hypothesized that insects can be used to achieve waste-free production cycles that enable more sustainable food production. The dried larvae are rich in protein (Spranghers et al., 2017) and can be used as supplements for animal rearing and aquaculture (Liu et al., 2017). However, the flexibility of the feed leads to challenges in process planning—both in the larval and fly stage—as the development speed, fly life span, egg production potential, etc. depend on it. While substantial efforts have been directed towards understanding the rearing process of the larvae (Bava et al., 2019; Padmanabha et al., 2020; Yakti et al., 2022), it is noteworthy that the reproductive processes of matured flies have not received the same degree of attention.

The production of eggs and young larvae often imposes a bottleneck on the maximum rearing capacities of a production site (Klüber et al., 2023). In standard practice, the flies will often sit idly in their cages without mating, wasting precious resources. However, the mating and oviposition process can be influenced through various controllable variables, such as light or temperature (Tomberlin and Sheppard, 2002; Zhang et al., 2010). At the same time, unnecessary movement and stress will drain the flies’ energy reserves, resulting in reduced life spans and fecundity (eggs per female). Optimizing egg production with minimized stress and energy costs while maximizing egg output necessitates a systematic control approach.

In this study, we present a process-control-oriented model designed to automate environment stimulation and to optimize the egg production process of the BSF. First, the fly life cycle, the mating process, and the egg production were analyzed and abstracted into mathematical models. The impact of control variables, including temperature and light, was systematically modeled. Model parameters were fitted to data available in literature and the model was tested for plausibility. Subsequently, a time-varying optimal control sequence for light, and temperature was computed for various scenarios. Simulation results were compared to a standard approach in which environmental conditions were kept at constant levels and the advantages of optimal control in egg production were highlighted. This work addresses issues in rearing efficiency, and process automation, thus improving economic viability of insect protein production.

## 2 Materials and methods

### 2.1 Modeling fly life cycle, mating, egg production, and death

**FIGURE 1:**
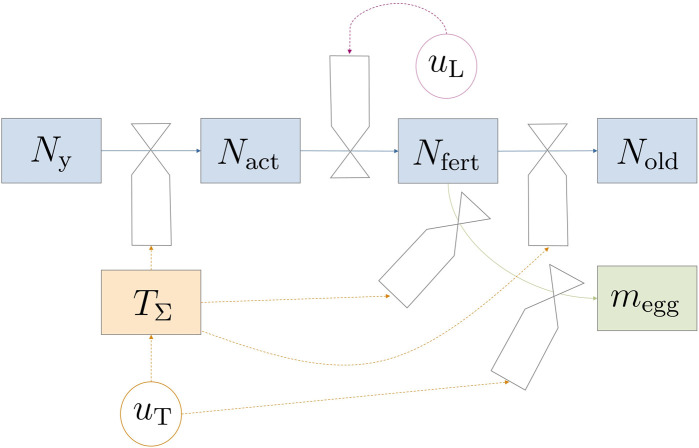
Forrester diagram illustrating the model dynamics. Circular valves labeled *u* represent controllable variables. The transition between life stages is predominantly influenced indirectly by temperature (*T*_Σ_) through *u*_T_, except for mating and subsequent fertilization, which are regulated by light *u*_L_.

This section introduces a comprehensive mathematical model for the adult flies’ life cycle from eclosion to adulthood while also considering oviposition processes. Controllable environmental variables such as light and temperature were incorporated, in recognition of their influential roles in the process. Figure 1 depicts the model dynamics in a forrester diagram. The parts of the model—fly life cycle and life stage dynamics, egg production, survival on energy reserves, and environmental impact factors—were derived individually and would be combined in Section 2.1.4. Model equations were mostly formulated in a mechanistic way to allow for physical interpretation of equations. A continuous state model that works with population averages was used since data for modeling probabilistic and discrete events (like individual fly death) are scarce. Additionally, the chosen modeling approach greatly reduces complexity for simulation and optimization.

The considered reproduction setup was a breeding cage with a certain number of male and female pupae placed within. Gobbi et al. (2013) reported a female bias in *Hermetia*; however, male flies can mate with multiple females and therefore no limiting effects would be observed in such unevenly distributed populations. While a percentage of flies could have been infertile or have life-threatening deformations, e.g., wing damage during emergence, such events were neglected and not included in the model. Under the previous assumptions, only the number of female flies *N* was relevant for egg production. Hence, male flies were not modeled, which reduced model complexity without losing plausibility (or predictive capabilities). At harvest, eggs are collected and taken out of the system; no new pupae or flies are introduced during a reproduction cycle. The basic structure for the adult fly life cycle is a life-stage model. The four life stages are introduced in the following paragraph.

#### 2.1.1 Fly life stages and life stage dynamics

The process starts when pupae are introduced into the breeding cage and ends either when all flies are dead or when the operator decides that too few eggs are produced. The four fly life stages are ‘young’, ‘active’, ‘fertilized’, and ‘old’. Flies are categorized as ‘young’ (*N*_y_) from the time of their introduction to the system as pupae and continue to be classified as such even after hatching. This classification extends throughout the period during which they unfold their wings, and undergo exoskeleton hardening persisting until mating occurs. After the young fly stage comes the sexually ‘active’ stage *N*_act_, where flies actively search for mating partners. A fraction of the population then has a chance to become fertilized, *N*_fert_. Fertilized flies actively contribute to the egg production process, and once they have completed oviposition, they transition into the ‘old’ category (*N*_old_).

The pupae placed in the breeding cage do not have the exact same life history, e.g., age, genetics, etc. While, for the individual fly, transition to the next life stage happens instantaneously, for the fly population in the breeding cage—due to their variations in life history and stochastics during mating—this is a gradual process. The rate of change was modeled as an ordinary differential equation (ODE)

$${\dot{N}}_{\text{y}}=-k_{\text{y}2\text{act}}N_{\text{y}}-k_{\mu ,N}\mu N_{\text{y}},$$ (1)

in which the dot notation was used to represent derivatives with respect to time. Parameter values for *k*_y2act_ can be found in Table 1 and *k*_*μ*,N_ = 1 d^−1^. The dying rate *μ* is introduced in the next section.

**TABLE 1:** List of parameters with source of data used for parameter fitting. Aiming for consistency across various findings, data from multiple authors were used for the development sums. When using data from (Chia et al., 2018), only data in the range of 20–37°C were used. Refer to Section 3.1.1 for more details.

Category	Parameters			Data from
life stage dynamics	*k*_y2act_ 0.28 d^−1^	*k*_act2fert_ 0.62 d^−1^	*k*_fert2old_ 0.55 d^−1^	Nakamura et al. (2016)
development sums	*k*_TΣmin_ 12°C	*k*_Σy2act_ 28^◦^Cd	*k*_Σovi_ 56^◦^Cd	various
oviposition	*k*_ovi_ 5.9 mg d^−1^			Nakamura et al. (2016)
*μ* related	*k*_*μ*_ 0.0183 d^−1^	*k*_*μ*,old_ 2.14 d^−1^	*k*_*μ*,ovi_ 2.5	Nakamura et al. (2016), Chia et al. (2018)
light on mating chance	*a*_1_ 0.986	*a*_2_ 0.368 d h^−1^		Hoc et al. (2019)
temperature on fecundity	*k*_T,ovi,0_–3.17	*k*_T,ovi,1_ 0.286 K^−1^	*k*_T,ovi,2_–0.005 K^−2^	Chia et al. (2018)
temperature on *μ*	*k*_T,*μ*,0_ 3.13	*k*_T,*μ*,1_–0.166 K^−1^	*k*_T,*μ*,2_ 0.0033 K^−2^	Chia et al. (2018)

The flies undergo certain internal development processes which result in time delays between hatching and mating, and fertilization and oviposition (Tomberlin and Sheppard, 2002). Chia et al. (2018) have found that temperature strongly impacts the time from emergence to oviposition which suggests that both time and temperature should be considered for development and stage transitioning. For this, now introduced development sums *T*_Σ_

$${\dot{T}}_{\Sigma }=\max\left(0,T-k_{\text{T}\Sigma min}\right)$$

with *T* as the temperature in the breeding cage and *k*_TΣmin_ as a threshold temperature below which development halts. The transition rates would be only activated once a minimum amount of development sums (i.e., threshold values) had accumulated

$$\begin{array}{ll} f_{y2act}\left(T_{\Sigma }\right) & =sw\left(T_{\Sigma }-k_{\Sigma y2act}\right)k_{y2act}\\ f_{fert2old}\left(T_{\Sigma }\right) & =sw\left(T_{\Sigma }-k_{\Sigma ovi}\right)k_{fert2old}\\ f_{ovi}\left(T_{\Sigma }\right) & =sw\left(T_{\Sigma }-k_{\Sigma ovi}\right)k_{ovi} \end{array}$$

where ‘sw’ is a switch function defined as follows

$$sw\left(x\right)=\left\{\begin{array}{cc} 0\; & \text{if }x\leq 0\\ 1\; & \text{else } \end{array}\right.$$ (2)

with *k*_Σy2act_ and *k*_Σovi_ being threshold values for mating behavior and oviposition, respectively. See Section 2.3.1 and Supplementary Material for a smooth implementation of max and sw functions to improve numerics. The next sections discusses fly dying rate and egg production.

#### 2.1.2 Energy reserves, fly survival, and egg production

The diet during the larval stage allows the flies to accumulate energy reserves (Gobbi et al., 2013), predominantly in the form of the so-called fat body. These energy reserves play a crucial role in determining both the remaining life span and the potential for egg production in the flies (Hall and Gerhardt, 2002). As the energy reserves diminish, the dying rate *μ* would increase at a rate of *k*_*μ*_

$$\dot{\mu }=k_{\mu }.$$ (3)

When *μ* = 0, this means most reserves are used up, and the flies begin to starve, risking death. In reality, there may have been early deaths due to problems during emergence or hardening of the chitin carapace, or when trying to unfold their insect wings. However, this was unrelated to *μ* and the choice of environment conditions for egg production optimization.

Neglecting such premature deaths, there isa delay before the first fly deaths start occurring—depending on initial weight and breeding cage setup, e.g., temperature and availability of nourishing fluids (Nakamura et al., 2016; Bertinetti et al., 2019; Macavei et al., 2020). One approach to model this phenomenon, similar to how changes in behavior for mating and ovipositing were handled, would be to use switch functions and development sums. However, employing such methods could have compromised the mechanistic interpretability of the model when explaining how access to water or nutrient-rich fluids impacted fly lifespan. Alternatively, a more interpretable method would involve working with the initial value *μ*(*t* = 0) = *μ*_0_. While *μ* was initially introduced as the dying rate, an alternative interpretation would be to view it as the proportion of energy reserves already consumed by the fly relative to a fly on the verge of starvation (i.e., *μ* = 0). Negative values of *μ* signify the presence of remaining energy reserves, while values greater than zero indicate that the flies are in a state of starvation (and consequently at risk of dying). It is important to note that *μ* is an abstract variable and cannot be directly measured. However, its effects can be readily observed by monitoring when and how many flies perish. The initial value *μ*_0_ is influenced by the wellbeing and accumulated reserves (i.e., weight) during the larval stage (see Section 2.2 for our parameter fitting approach).

As seen in Eq. 1, flies die at a rate of −*k*_*μ*,N_ *μN*. However, negative values of *μ* would lead to an increase of population, which cannot happen in reality. That is why the dying term −*k*_*μ*,N_ *μN* needed be modified such that population would not increase for negative *μ*, i.e.,

$$-k_{\mu ,N}\max\left(0,\mu \right)N.$$ (4)

In the adult stage, due to their mouth physiology, the flies can only consume fluids and no solids (Bruno et al., 2019). Consuming fluids like water, nectar, or milk slows down decay of energy reserves and increases egg production (Bertinetti et al., 2019; Bruno et al., 2019; Klüber et al., 2023). To accommodate for feeding, Eq. 3 could be extended with a positive term that is added and therefore slows down the decay of energy reserves. However, the purpose of this model is to calculate dynamic trajectories for environmental factors. Choosing a fluid to constantly supply the flies with does not require such sophisticated methods. This concludes the description of the dynamics of *μ*.

As previously stated, *μ* influences the potential for egg production, which can be described with

$${\dot{m}}_{\text{e}}=\left(1-k_{\mu ,ovi}\mu \right)k_{\text{ovi}}N_{\text{fert}},$$ (5)

where *k*_ovi_ is a constant for egg production per fertilized fly and *k*_*μ*,ovi_ adjusts how the energy reserves impact egg production. It can be seen that bigger larvae and flies (i.e., negative *μ*_0_) result in higher egg mass. This also means that the less energy flies spend before ovipositing, the more eggs they can produce. In a study by Tomberlin and Sheppard (2002), it was hypothesized that females may reabsorb oocytes to maintain respiration, resulting in reduced egg clutch size. Keeping stress levels and unnecessary movement to a minimum enhances production potential.

Nakamura et al. (2016) found that flies that do not mate lived significantly longer, suggesting that the search for a mating partner and oviposition were the biggest energy drains for the flies. To model the flies dying faster after oviposition, the dying rate was multiplied by a factor *k*_*μ*,old_.

The upcoming section explores how environmental variables, including temperature and light, influence fly behavior.

#### 2.1.3 Factors influencing the life cycle of adult flies

Recall that the aim is optimization of BSF egg production by controlling certain environmental variables, as previously illustrated in Figure 1. The three most influential factors during the life of the fly are temperature, lighting, and access to nutrient rich liquids (Holmes et al., 2012; Chia et al., 2018; Bertinetti et al., 2019; Hoc et al., 2019; Nayak et al., 2024). Relative humidity of air impacts multiple life history traits, including development speed (Holmes et al., 2012). However, data on the effect of relative humidity on mating behavior were insufficient for modeling. Results of (Holmes et al., 2012) suggests that relative humidity should be kept sufficiently high and at a constant level. Thus, dynamic trajectories of humidity do not seem to have any optimization potential and will not be modeled.

**FIGURE 2:**
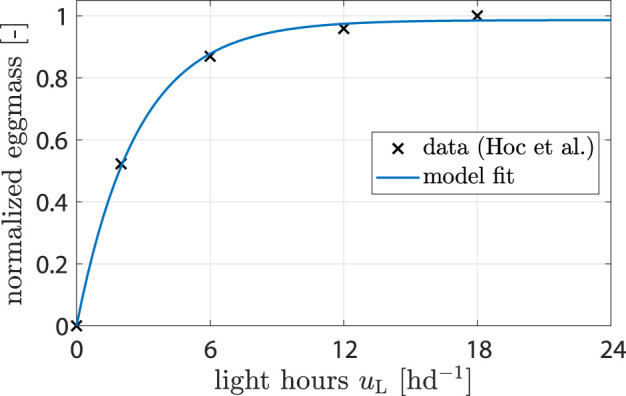
Data showing normalized egg mass from (Hoc et al., 2019). Model fit shows response of *ξ*_L_ (*u*_L_).

For the influence of light *ξ*_L_ the control variable is *u*_L_, i.e., light hours per day. Only white light was considered, and the effects of different wave lengths and light intensities were neglected. Hoc et al. (2019) found that more light hours per day increase the amount of eggs harvested. The reason is that the chance of finding a partner for mating is enhanced (Jones and Tomberlin, 2021). Data from (Hoc et al., 2019) were used to fit a model of the form

$$\xi_{\text{L}}\left(u_{\text{L}}\right)=a_{1}\left(1-\exp\left(-a_{2}u_{\text{L}}\right)\right),$$ (6)

where *a*_1_ and *a*_2_ are parameters. Parameter values were determined from data (see Figure 2 and Table 1; Section 2.2).

Temperature influences three properties of fly life: lifespan, fecundity, and life stage transition speed. The concept of development sums already encompasses the transition speed, as higher temperatures contribute to the accelerated accumulation of *T*_Σ_, resulting in earlier sexual activity and oviposition.

**FIGURE 3:**
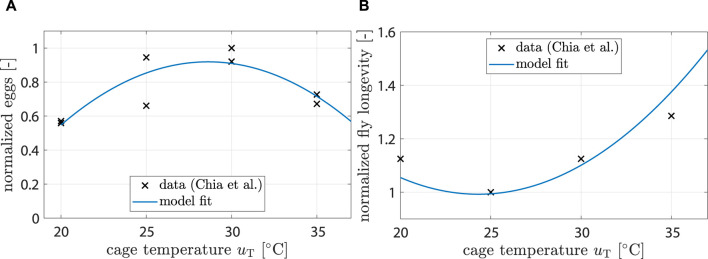
**(A)** Data showing normalized egg mass from (Chia et al., 2018). Model fit shows response of *ξ*_T,ovi_ (*u*_T_). **(B)** Data showing normalized fly life duration from (Chia et al., 2018). Model fit shows response of *ξ*_T,mu_ (*u*_T_).

Chia et al. (2018) found that fecundity (i.e., number of unfertilized eggs per female) is significantly affected by temperature. A 2^nd^ order polynomial was found to be a good fit:

$$\xi_{\text{T,ovi}}\left(u_{\text{T}}\right)=k_{\text{T,ovi,}2}u_{\text{T}}^{2}+k_{\text{T,ovi,}1}u_{\text{T}}+k_{\text{T,ovi,0}},$$ (7)

with *k*_T,ovi_ being the respective polynomial coefficients and *u*_T_ being the controllable temperature inside the breeding cage (see Figure 3A).

Chia et al. (2018) also found that longevity is significantly affected by temperature. A 2^nd^ order polynomial was found to be a good fit:

$$\xi_{\text{T,}\mu }\left(u_{\text{T}}\right)=k_{\text{T,}\mu ,2}u_{\text{T}}^{2}+k_{\text{T,}\mu ,1}u_{\text{T}}+k_{\text{T,}\mu ,0}.$$ (8)

Fitting results can be seen in Figure 3B.

#### 2.1.4 Combined model

The combined model includes the influence of *u*_T_ and *u*_L_ and the cross dynamics between *T*_Σ_ and *N*_*i*_ as well as *μ* and *m*_e_:

$${\dot{N}}_{\text{y}}=\;-f_{\text{y}2\text{act}}\left(T_{\Sigma }\right)N_{\text{y}}\;-k_{\mu ,N}\max\left(0,\mu \right)N_{\text{y}}$$ (9)

$${\dot{N}}_{\text{act}}=f_{\text{y}2\text{act}}\left(T_{\Sigma }\right)N_{\text{y}}\;-f_{\text{act}2\text{fert}}\xi_{\text{L}}\left(u_{\text{L}}\right)N_{\text{act}}\;-k_{\mu ,N}\max\left(0,\mu \right)N_{\text{act}}$$ (10)

$${\dot{N}}_{\text{fert}}=f_{\text{act}2\text{fert}}\xi_{\text{L}}\left(u_{\text{L}}\right)N_{\text{act}}\;-f_{\text{fert}2\text{old}}\left(T_{\Sigma }\right)N_{\text{fert}}\;-k_{\mu ,N}\max\left(0,\mu \right)N_{\text{fert}}$$ (11)

$${\dot{N}}_{\text{old}}=f_{\text{fert}2\text{old}}\left(T_{\Sigma }\right)N_{\text{fert}}\;-k_{\mu ,old}\max\left(0,\mu \right)N_{\text{old}}$$ (12)

and.

$${\dot{T}}_{\Sigma }=\max\left(0,T-k_{\text{T}\Sigma min}\right)$$ (13)

$$\dot{\mu }=\xi_{\text{T,}\mu }\left(u_{\text{T}}\right)k_{\mu }$$ (14)

$${\dot{m}}_{\text{e}}=\left(1-k_{\mu ,ovi}\mu \right)\xi_{\text{T,ovi}}\left(u_{\text{T}}\right)f_{\text{ovi}}\left(T_{\Sigma }\right)N_{\text{fert}}.$$ (15)

### 2.2 Parameter fitting

Parameters were fitted to data using functions from MATLAB (r2022a) on a standard desktop PC. Initially, development sum dependent stage transition parameters were identified using linear regression. Next, base parameters (i.e., *ξ*_T_ = *ξ*_L_ = 1) for life stage transition dynamics, oviposition, and dying rate were identified by minimizing the difference between simulation results and data from literature using *lsqcurvefit*. *Polyfit* was used to fit a parabola for *ξ*_T,ovi_ and *lsqnonlin* to fit *ξ*_L_. Finally, similar to the base parameters, *ξ*_T,*μ*_ was identified by minimizing the difference between simulation results and literature data. The source of data used for each identification step is described in Table 1, and fitting results are shown in Section 3.1.1 and Figure 2 as well as 3.

The starting value for dying rate *μ*_0_ could not be determined through measurements but instead had to be chosen in accordance with model assumptions and boundary conditions. As was explained during modeling, *μ*_0_ is negative because there is a delay between emergence and first death. Next, we know that—neglecting deaths due to accidents during emergence—first flies start dying after ≈7 days (Nakamura et al., 2016). Lastly, data from (Nakamura et al., 2016) indicated that the dying rate after 14 days should be *μ* ≈ 0.15 days^−1^. With these three conditions, the starting value was chosen to be −0.1, and parameters were fit accordingly.

### 2.3 Simulation and optimization of egg production

In this section, the framework for simulation and optimization of the egg production process is described. This includes explanation of the method, process constraints, and other criteria. Simulations, parameter fitting, and optimization were all performed in MATLAB (r2022a) (The MathWorks, 2022).

#### 2.3.1 Simulation framework

Eqs 9–15 describe a system of (non-stiff ordinary) differential equations. Integrator *cvodes* from CasADi Matlab toolbox (Andersson et al., 2019) was used for numeric integration using the backward differentiation formula (BDF). Step size was 1 h, and simulation time was 14 d to reflect typical production times. The sw and max functions used in, e.g., Eqs 2, 4, respectively, caused numerical difficulties during optimization, which is why they were instead implemented through a tangent hyperbole function $\left(\frac{1}{2}+\frac{1}{2}\tanh\left(kx\right)\right)$ , where *x* is a state variable and *k* was chosen to be large enough to make the tanh steep (see Supplementary Figures S1, S2).

For the population *N*_*i*_, all states except *N*_y_ were zero, since pupae are only introduced to the system in the beginning. The number of young flies at the beginning was set to 50 to ensure simulation results were comparable with data from (Nakamura et al., 2016). Egg mass in the beginning was zero. Development sums were zero as well, since, after emergence, a new life stage starts, and it was assumed that temperature history during pupae stage would not influence the life of the fly. The starting value for dying rate was *μ*_0_ = −0.1 as explained in Section 2.2. Standard environment conditions were taken from Nakamura et al. (2016) with *u*_T_ = 25°C and *u*_L_ = 16 h d^−1^.

#### 2.3.2 Optimization approach, framework, and variables

The model detailed in the preceding sections was formulated with the objective of optimizing egg production. This was achieved through the manipulation of environmental process parameters, specifically temperature and daily light exposure hours. A model-based optimization approach was used to find optimal trajectories for *u*_T_ and *u*_L_. The CasADi Matlab toolbox was used for the implementation of the optimization algorithm (Andersson et al., 2019). Lower and upper bounds for *u*_T_ were chosen as 20°C and 37°C, respectively, under consideration of data from (Chia et al., 2018). The upper bound for *u*_L_ was 24 h d^−1^, and the lower bound was chosen as 2 h d^−1^ as the minimum stimulus required to keep flies alive (Nakamura et al., 2016; Hoc et al., 2019). Interior point optimizer (*ipopt*) was used as the solver, which utilizes gradients and Hessians obtained through symbolic and automatic differentiation (Wächter and Biegler, 2006). Input trajectories were discretized into 1 hour intervals. Time horizon for optimization was *t*_end_ = 14 d to reflect typical production times.

#### 2.3.3 Optimization aim

To maximize egg output *m*_e_ with minimal effort, i.e., minimize input ***u*** ∈ {*u*_T_, *u*_L_}, the optimization problem was formulated as follows

$$\begin{array}{cc} \underset{u}{\min}\;\int_{0}^{t_{\text{end}}} & -Qm_{\text{e}}+u^{⊤}Ru\;dt\\ s.t.\; & dynamic\;model\;Eq.\;\left(9\right)\;to\;\left(15\right)\\ & 20^{◦}\text{C}\leq u_{\text{T}}\leq 37^{◦}\text{C}\\ & 2\;\text{h}\;{\text{d}}^{-1}\leq u_{\text{L}}\leq 24\;\text{h}\;{\text{d}}^{-1} \end{array}$$ (16)

with weights ***R*** and ***Q***. A common approach for choosing the weights is to abstract them into money, i.e., costs per kilo watt hour and profit per milligram egg mass. However, since those prices vary vastly by countries, a different approach was chosen here. Note that the optimization method and cost function were formulated in a generalized manner, allowing for easy adaptation if economic parameters are available. Instead, weights were chosen such that both *m*_e_ and ***u*** have similar importance. From simulations, we know the expected value of *m*_e_ (*t*_end_) ≈ 300 mg, while ***u*** ranges between low single to double digits. Thus, for equal weighing, the ratio should be $R/Q\overset{!}{\approx }10$ . Based on systematic testing, we chose ***Q*** = 12 mg^−1^ and ***R*** = 100*I*_2_ (units omitted).

The cost function awarded egg mass at every time step instead of only final mass at the end of production cycle *t*_end_. That way, the optimization calculated a trajectory that balances maximum output and production speed.

## 3 Results and discussion

Parameter fitting results are explained, and the plausibility of the theoretical model is assessed. Afterwards, optimization is evaluated in various scenarios.

### 3.1 Simulation studies and comparison to literature data

Model performance was evaluated by comparing simulation results with data from literature. However, the scarcity of available data and substantial variations in experimental setups among different authors posed challenges for parameter identification and model validation. Differences during the larval rearing stage can especially influence adult fly performance, including longevity and fecundity. However, we assumed that, regardless of life history before pupation, the flies’ responses to environmental variables are the same. Thus, computed control trajectories would improve egg production even under parameter uncertainties.

#### 3.1.1 Plausibility of environment impact

**FIGURE 4:**
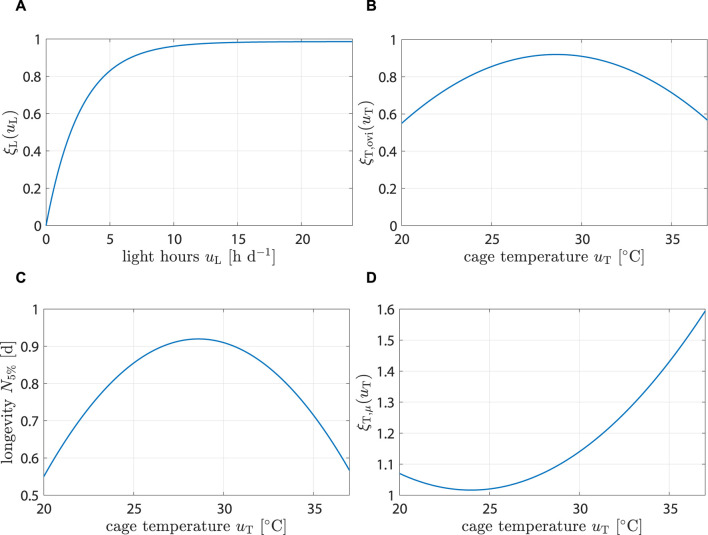
Change in fly performance in dependence of environment parameters. **(A)** Egg production is reduced by 10% when going from 16 to 6 h d^−1^. However, at 2 h d^−1^, egg production is reduced to 51% of maximum value. **(B)** Fly fecundity peaks at 28.6°C and is reduced by 23% at 35°C. **(C)** Longevity (i.e., when population reaches 5% of its starting value). is longest at 24°C and is reduced by 3.5 d (≈29%) at 35°C. **(D)** Fly dying rate increases most slowly at 24°C, but grows 42% faster at 35°C.

Data from Hoc et al. (2019) were utilized to calibrate the light impact component from Eq. 6 (see Figure 4A). The model predicted that egg production would be reduced by 19% when *u*_L_ is decreased from 16 to 6 h d^−1^ and by 53% from 16 to 2 h d^−1^. These predictions aligned well with the provided data and were consistent with observations reported by Liu et al. (2021).

For the impact of temperature on fecundity, Eq. 7, data from (Chia et al., 2018) were used (see Figure 4B). The model predicted highest fecundity at 28.6°C and a significant drop for temperatures above 35°C, which agreed well with (Chia et al., 2018; Shumo et al., 2019).

No data were found that describe the impact of temperature on the time period from emergence until first mating behavior occurs, but (Tomberlin and Sheppard, 2002) reported this to be approximately 2 days. Due to the lack of data, parameter *k*_Σy2act_ was decided to be half of *k*_Σovi_ (preoviposition period). Chia et al. (2018) investigated the influence of temperature on the preoviposition period; however, their findings conflicted with other authors. While Chia et al. (2018) found mean preoviposition time to be above 9 days at both 25 and 30°C, other authors report periods of 3–5 days (Tomberlin and Sheppard, 2002; Nakamura et al., 2016; Heussler et al., 2018; Hoc et al., 2019; Liu et al., 2021). We chose the parameters in such a way that the model produced plausible results. Little data was found on the exact minimum required temperature *k*_TΣmin_ for development, but (Chia et al., 2018) reported that development still occurred at 15°C. The parameter *k*_TΣmin_ was chosen as 12°C such that the model produced plausible results.

Fitting parameters for Eq. 8 was challenging since *μ* cannot be directly measured. The parameters had to be indirectly inferred by observing longevity at different temperatures. Experiments conducted by Chia et al. (2018) provided relevant data; however, their findings conflicted with observations from other authors. For instance, while Nakamura et al. (2016) reported longevity to be around 18 d at 25°C (with distilled water provided to flies), data from Chia et al. (2018) indicated a longevity of around 14 days at the same temperature (with sugar water provided)—a notable discrepancy. It is important to note that mating behavior and oviposition also indirectly influence longevity. Higher temperatures accelerated the development time, causing flies to lay eggs earlier—a highly energy-consuming process that can reduce the overall lifespan. Nakamura et al. (2016) reported that female flies in separated colonies lived up to 47.6 d when provided with sugar water. It cannot be distinguished if decreases in longevity at higher temperatures were due to earlier oviposition or due to increased energy consumption from higher fly activity. Experiment setups between authors differed greatly; feeding during larval rearing stage, final pupae weight, population genetics, liquid feed provided during adult stage, etc. all impact longevity, which made finding general parameters that robustly predict longevity impossible. Parameters were chosen again for plausibility (see Figures 4C,D).

#### 3.1.2 Simulation studies

##### 3.1.2.1 Standard conditions

**FIGURE 5:**
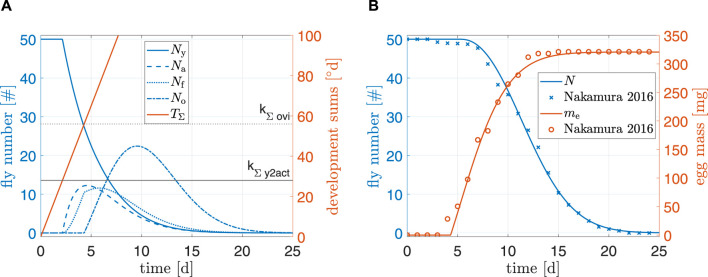
**(A)** Simulation results and **(B)** comparison to data from Nakamura et al. (2016). Simulation starting values are *N*_y0_ = 50 and *μ*_0_ = −0.1.

Figure 5 shows simulation results of the model under standard conditions. For *N* and *m*_e_, root mean squared errors were 1.1 and 8.6 mg, respectively. Furthermore, a visual comparison of both total fly population and egg production to data from (Nakamura et al., 2016) indicated a reasonably good fit. Our model did not predict individual flies dying after only 3 days; it is possible that individual pupae in the experiments of Nakamura et al. (2016) suffered from malnutrition, were sick, or damaged themselves during emergence. Final egg mass of the simulation overshot data by 0.15%. Egg production in the beginning seemed to be delayed in comparison to the data; the reason might be because the model for preoviposition time (*k*_Σ,ovi_) was fitted to data from a multitude of authors. This delay can also explain why egg mass in experimental data reached its peak faster than model predictions and why model predictions of *N* were slightly off around day 7 (earlier oviposition results earlier deaths). Overall, the simulation results indicated a plausible model for standard conditions.

##### 3.1.2.2 Non-standard environment conditions

**FIGURE 6:**
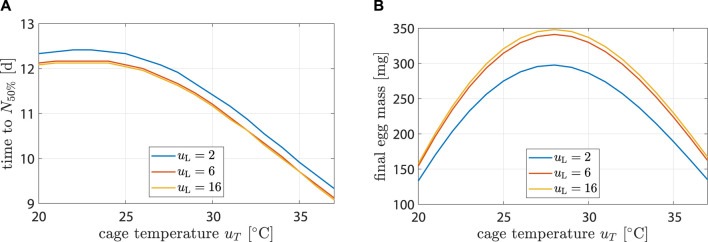
Life history characteristics as a function of environmental conditions. Plot **(A)** shows time until population is halved while **(B)** hows egg mass after 14 d.

The model was controlled dynamically through environment variables *u*_L_ and *u*_T_. To better understand how the model behaved in response to different inputs, see simulation results in Figure 6 or Supplementary Figure S3. Egg production and longevity were investigated at three different light input levels and over a range of temperatures.

Highest longevity was found at 22°C. At *u*_L_ = 2 h d^−1^, longevity decreased by 25% (12.4 d–9.3 d) from *u*_T_ = 22°C to 37°C. At *u*_T_ = 22°C longevity decreased by 2% (12.4 d–12.1 d) from *u*_L_ = 2 h d^−1^ to 16 h d^−1^. Impact of *u*_L_ on longevity was negligible. This agreed with (Liu et al., 2021), who found that the survival rate after 10 d did not differ significantly with changes in light regime.

Highest egg production occurred at *u*_L_ = 16 h d^−1^ and *u*_T_ = 28°C with 348 mg. It decreased by 52% (167 mg) and 54% (157 mg) at 37°C and 20°C, respectively. At *u*_T_ = 28°C, egg production decreased by 2% (348 mg–341 mg) from *u*_L_ = 16 h d^−1^ to 6 h d^−1^ and by 17% (348 mg–298 mg) from *u*_L_ = 16 h d^−1^ to 2 h d^−1^. Results were compatible with the findings of (Hoc et al., 2019).

First oviposition occurred after 2.46 d at 35°C, while at 30 and 25°C it occurred after 3.1 and 4.3 d, respectively. This agreed well with data from (Bertinetti et al., 2019; Binsin et al., 2023).

In the range of reasonable inputs, the model delivered plausible predictions and was sensitive to changes in control variables. Note how longevity, fecundity, and development speed all have their maximum at different temperatures. These differences were the reason why temperature trajectories have high potential for egg production optimization compared to constant temperatures. The optimization potential is explored in the following section.

### 3.2 Results of optimal control

#### 3.2.1 Optimization potential

Higher temperature increased the development speed of the flies, i.e., oviposition occurs sooner, which is beneficial in industrial production. Simultaneously, increased fecundity is observed at higher temperatures. This came at the price of higher operation costs and accelerated dying rate. However, keeping dying rate *μ* low increased egg production. This resulted in conflicting requirements for *T*_Σ_, *ξ*_T,mu_, and *ξ*_T,ovi_ with concern to *u*_T_.

Changing *u*_L_ did nothing while there were no active flies, and, therefore, light hours should be kept short at the beginning of the reproduction cycle.

Certain assumptions and simplifications were made while modeling. As previously mentioned, relative humidity, choice of liquid food supplements, population density, and light quality parameters such as wave length spectrum and intensity can impact the egg production process (Klüber et al., 2020; Nayak et al., 2024). However, there are—to our knowledge—no dynamic effects to these process parameters. It thus sufficed to make a choice at the beginning and to keep those parameters constant.

Another simplification was that, while fecundity (number of eggs laid per female) was modeled, fertility (number of fertilized eggs) and hatchability (portion of fertilized eggs that survive and become larvae) were neglected. It is important to recognize that an increase in fecundity might not necessarily lead to a proportional increase in the number of hatched larvae if optimal temperatures for fecundity, fertility, and hatchability differ. This implies that optimizing the process solely based on fecundity may yield suboptimal results for the reproduction of larvae.

Another necessary simplification was that dynamic effects (i.e., long term effects) had to be neglected due to lack of information. For instance, exposure to high temperatures during the early stage (for accelerated development) might influence fecundity in the fertile stage. Additionally, there is a question of defining temperature in this context—whether it refers to the currently measured temperature, the average temperature, or the highest temperature since emergence.

#### 3.2.2 Standard scenario

**FIGURE 7:**
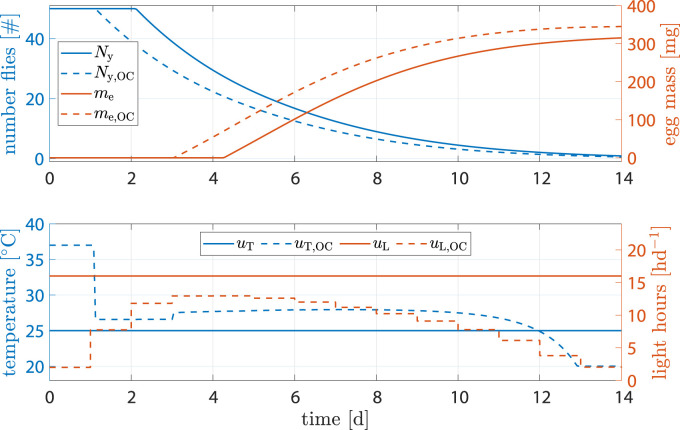
State and input trajectories for benchmark scenario *versus* OC with ***R*** = 100*I*_2_ (units omitted). Benchmark inputs are constant 16 h d^−1^ light period and 25°C.

The benchmark scenario was chosen similar to (Nakamura et al., 2016) with 16 h d^−1^ and constant 25°C. Process optimization was performed according to Eq. 16. Resulting state trajectories can be seen in Figure 7.

Comparison of states shows that, while fly population declined faster, the amount of eggs per cycle increased by 9.4% after 14 d (benchmark 315.2 mg *versus* optimized 345.2 mg). The shorter life cycle was a result of faster transition between life stages due to higher temperature at early stages. Flies reached sexual maturity faster but also burn their energy reserves faster. Shorter life cycles, however, mean that breeding cages can be refilled more often, resulting in more cycles and consequently higher egg production per time.

A production cycle abort criteria of 90% of predicted maximum egg mass was defined. With that, Table 2 shows results on how much production could be improved through optimization. While the total energy invested into heating was 3.7% higher, light input was reduced by 41.4%, cycle time was reduced by 1 d and egg production was increased by 9.4%.

**TABLE 2:** Accumulated inputs and final egg mass when abort criterium of *m*_egg_ > 0.9*m*_e_ (*t*_end_) was violated.

	Σ*u*_T_	Σ*u*_L (h)_	Time (d)	*m*_e (mg)_
benchmark	270^◦^Cd	173	10.9	284
optimization	281^◦^Cd	101	9.9	311

#### 3.2.3 High penality for inputs

**FIGURE 8:**
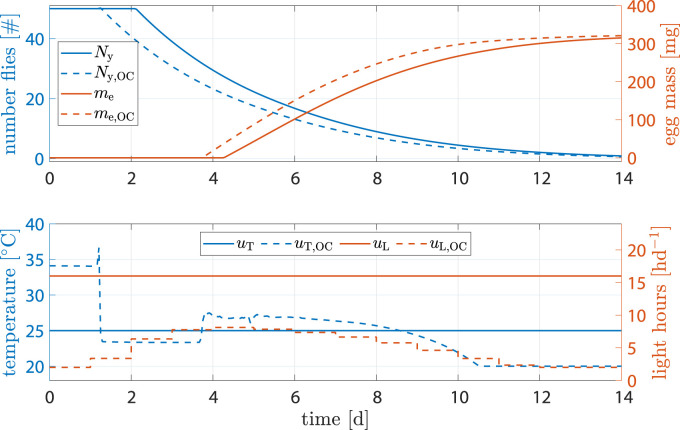
State and input trajectories for benchmark scenario *versus* OC with ***R*** = 1000*I*_2_ (units omitted). Benchmark inputs are constant 16 h d^−1^ light period and 25°C.

The benchmark scenario was chosen similarly as before with 16 h d^−1^ and constant 25°C. However, input weights were set to *R*_11_ = *R*_22_ = 1000. The reasoning was that light and heating can be rather costly and leave high CO_2_ footprints; a reduction of inputs ***u*** is of interest. Figure 8 shows the results. Egg mass after abort criteria in the benchmark was 284.2 mg *versus* 289 mg, an increase of 1.7%. Heat input was decreased by −7.33% and light input by −66.6%. Production time was decreased by 1.3 d.

#### 3.2.4 Light control only

**FIGURE 9:**
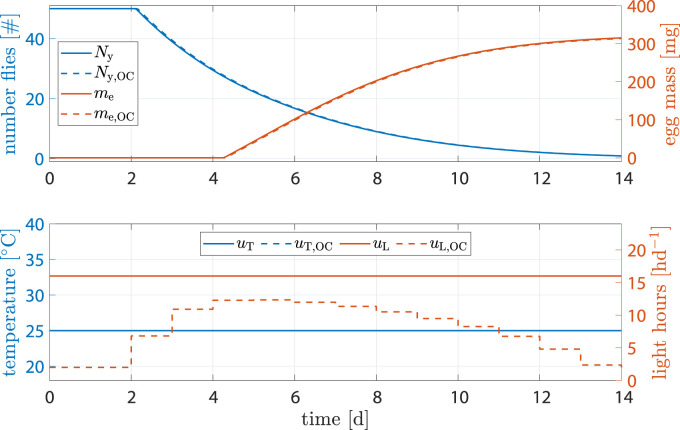
State and input trajectories for benchmark scenario *versus* OC where only light is controlled. Benchmark inputs are constant 16 h d^−1^ light period and 25°C.

Egg production facilities may not allow for precise control of temperature, or breeding may happen in big halls with multiple unsynchronized breeding cages. In such cases, dynamic control of temperature is no longer an option, but optimization of light stimulation still is. Simulations showed that, while impact on egg output and time related performance parameters was negligible, light input could be reduced by −44% when compared to the benchmark scenario (see Figure 9).

#### 3.2.5 Delay production to avoid weekends and holidays

Another plausible scenario, especially in batch setups, involved intentionally delaying oviposition. The process of egg collection typically involves manual labor. If the initiation of the oviposition phase aligns with weekends and holidays, there might be up to 4 days during which eggs are not collected. The designated areas for oviposition, usually specialized units designed for easy egg extraction, may become full, prompting the flies to avoid these areas and instead lay eggs in locations where collection is less convenient.

**FIGURE 10:**
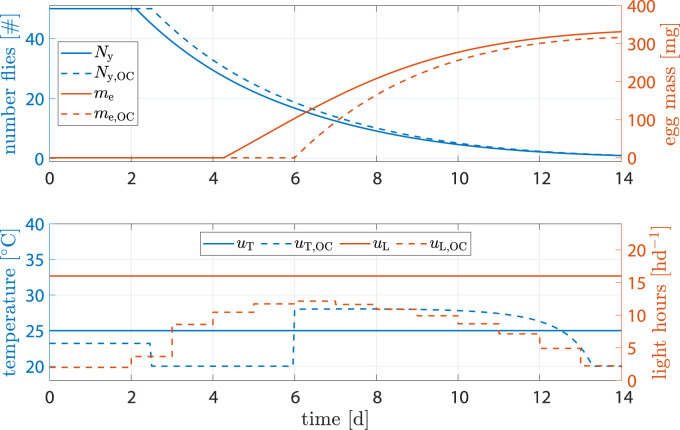
State and input trajectories for benchmark scenario *versus* OC where production is intentionally delayed. Benchmark inputs are constant 16 h d^−1^ light period and 25°C.

To address this issue, we utilized our model-based control to formulate a strategy capable of intentionally delaying oviposition by 2 days. This action aimed to potentially minimize the overlap between the egg collection window and labor-free days. For that, we introduced a constraint that ensured *T*_Σ_(*t* = 6) ≤ *k*_Σovi_. Optimization results can be seen in Figure 10. While the standard approach seemingly produced 4.5% more egg mass, not all of them could be harvested, as previously described. Assuming that one-third of the eggs oviposited before day 6 could not be collected, amount of eggs harvested using optimal control was actually 10.6% higher.

## 4 Conclusion and outlook

We have developed a dynamic control model that describes the life cycle and oviposition processes of *Hermetia* flies as a function of environmental factors. Dynamic models are in general useful tools for life cycle assessment and process monitoring, where comparison of measurements to model prediction may help in detecting possible faults. Other uses are process analysis through simulation studies and—as was showcased in this work—process optimization and control. During the review of literature, one thing became clear: there is a lack of information on dynamic effects of *u*_L_ and *u*_T_ on *Hermetia* flies. Most experiments in literature are conducted at near constant parameters. One, however, wants to dynamically control the environment which leads to the question of whether certain effects have a ‘memory’. For example, high temperatures during the young stage results in faster life stage transition to the active stage. However, fecundity is impaired by too high of a temperature, so temperature is reduced when first ovipositing occurs. According to the model, no problems would arise and one could benefit from fast development and high egg output simultaneously. But in reality, temperature might already affect fecundity during the time between becoming fertilized and oviposition. If the flies’ life processes have something similar to a ‘memory’ in regards to environmental parameters, it raises the question on just what the meaning of parameters such as temperature is: the current temperature, highest temperature since emergence, or some average temperature? Due to lack of data, such effects could not be considered during modeling. The absence of proper modeling for long-term effects raises concerns regarding the optimization algorithm potentially generating trajectories that appear optimal within the model but could be detrimental in real-world application. Approaches to address this challenge are the incorporation of process knowledge not only into the model itself but also into the process constraints during optimization or the extension of the model.

Possible extensions of the model may involve the identification of additional process control variables. For instance, studies may explore how airborne time of flies could be influenced, as this potentially increases the chances of finding a mating partner. Inputs such as sound, light flashes, or air jets represent potential variables for investigation. Environmental factors such as temperature and humidity have an effect on fertility and hatchability—processes which could also be included in the model.

The plausibility of the model was examined and tested against literature data, reaffirming that the model dynamics evolve in a realistic and plausible manner. We then used the model to calculate input trajectories to optimize the egg production process in various scenarios. Compared to a benchmark scenario, in our simulation study, the optimized process was able to generate more egg mass (9.4%) in less time (1 day faster) at reasonable costs (−41% *u*_L_ and +3.7% *u*_T_). Overall, we could show the versatility and usefulness of the model and optimal control in various scenarios. A possible extension to the optimization is to parameterize weights ***Q*** and ***R*** based on economic factors—allowing for direct economical evaluation of the optimization.

In our forthcoming work, we aim to address a critical aspect—parameter uncertainties, with a specific focus on *μ*_0_. Since *μ* cannot be measured, the starting condition *μ*_0_ was chosen without a strong data basis. It was tested how the model reacts to a parameter uncertainty of 50%—which can also be interpreted as very well fed larvae/pupae (see Supplementary Figure S4). A change of 50% in the initial value results in a 46% increase in time until the first fly dies and in an increase of 24% in produced egg mass. Georgescu et al. (2020) have found that female weight impacts fly egg output. This shows that the reactions of the model to parameter changes in *μ*_0_ are plausible. However, it also shows that the model is rather sensitive to this parameter and a fitting choice for *μ*_0_ (which cannot be directly measured) is crucial and subject to future experimental work.

## Data Availability

Publicly available datasets were analyzed in this study. This data can be found here: https://doi.org/10.1371/journal.pone.0216160.s001—Hoc et al. (2019) light on mating https://doi.org/10.1007/s13355-015-0376-1—Nakamura et al. (2016) data on population survival https://doi.pangaea.de/10.1594/PANGAEA.895274—Chia et al. (2018) on temperature related data.
