# Supplementary material for: Model-based process optimization of black soldier fly egg production
Source: Front Bioeng Biotechnol. 2024 May 22;12:1404776. doi: 10.3389/fbioe.2024.1404776 (PMC11150853; doi:10.3389/fbioe.2024.1404776)
Supplement: Supplementary file 1 [file DataSheet1.pdf]

Supplementary Material

1 FIGURES

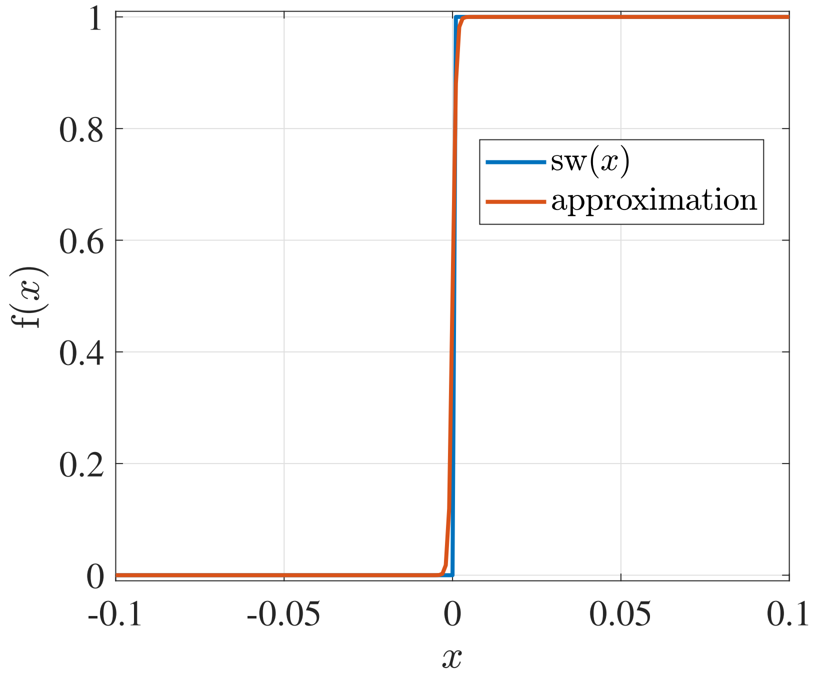

Figure S1. Function $\mathrm{sw}(x)$ is approximated using $(\frac{1}{2} + \frac{1}{2}\tanh(kx))$ with $k = 1000$. While it is inaccurate around 0, this implementation eliminates numerical issues during optimization.

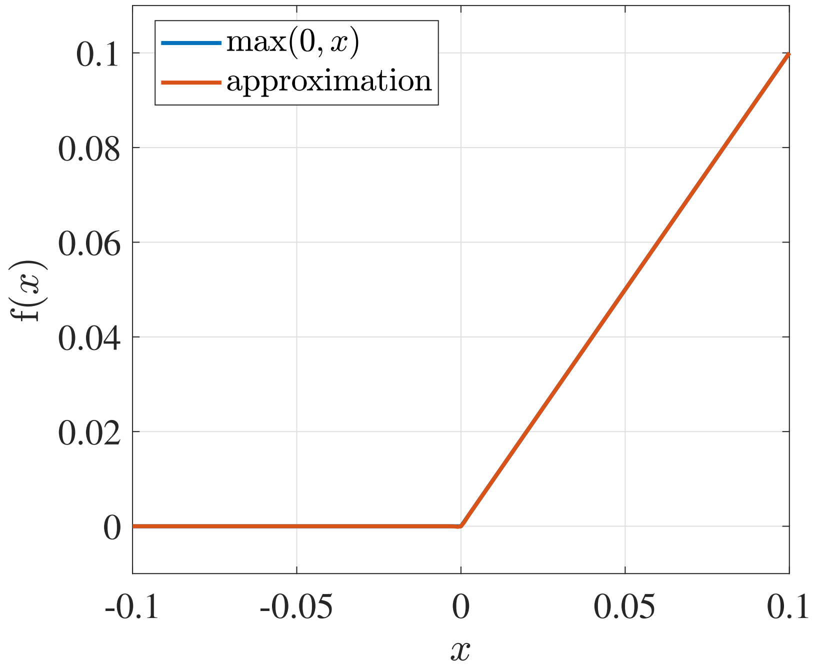

Figure S2. Function $\max(0, x)$ is approximated using $(\frac{1}{2} + \frac{1}{2}\tanh(kx))x$ with $k = 1000$. While it is inaccurate around 0, this implementation eliminates numerical issues during optimization.

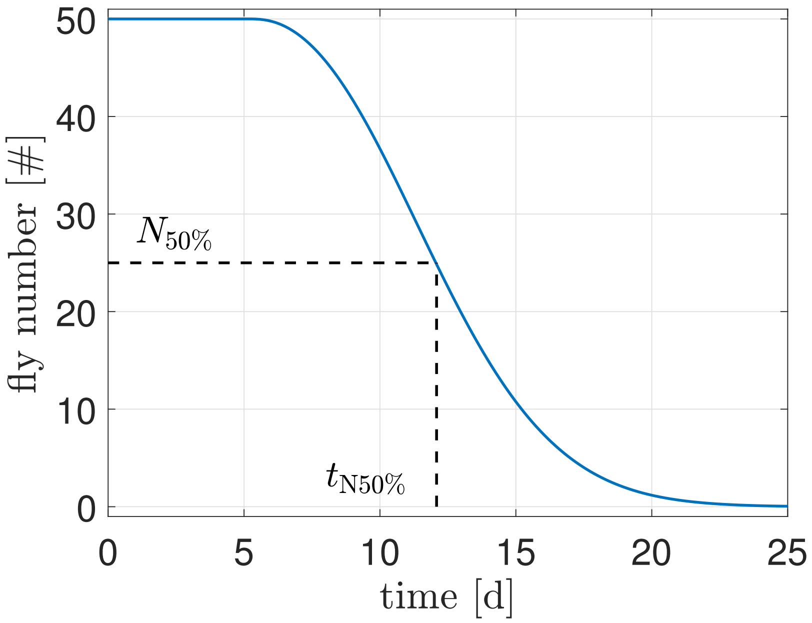

Figure S3. Fly population over time. The time it takes for the starting population to be halved, i.e. $N_{50\%}$, is $t_{\mathrm{N50\%}}$.

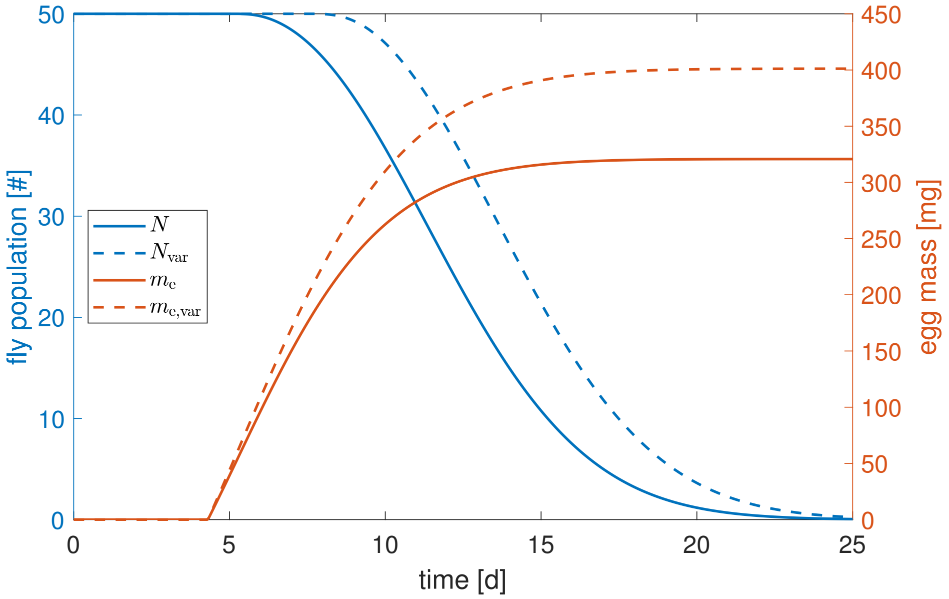

Figure S4. Simulation results of the model under standard conditions with $\mu_0 = -0.1$ versus a variation in initial conditions $\mu_{0,\mathrm{var}} = -0.15$. A change of 50 % in initial value results in an increase of 46 % in time until first fly dies and an increase of 24 % in produced egg mass.
